# Exercise Affects Cardiopulmonary Function in Patients with Chronic Kidney Disease: A Meta-Analysis

**DOI:** 10.1155/2017/6405797

**Published:** 2017-08-28

**Authors:** Hongchang Yang, Xueping Wu, Min Wang

**Affiliations:** ^1^Physical Education Department, Hohai University, Nanjing 210098, China; ^2^School of Physical Education and Coaching, Shanghai University of Sport, Shanghai 200438, China; ^3^Shanghai Key Lab of Human Performance, Shanghai University of Sport, Shanghai 200438, China; ^4^Scientific Research Department, Shanghai University of Sport, Shanghai 200438, China; ^5^Physical Education Department, Shanghai University of Finance and Economics, Shanghai 200433, China; ^6^School of Kinesiology, Shanghai University of Sport, Shanghai 200438, China

## Abstract

This study aimed to comprehensively assess the effects of exercise on cardiopulmonary function indices in patients with chronic kidney disease (CKD). A literature review was performed by searching literatures in PubMed and Embase before June 2016. Studies were selected based on predefined inclusion and exclusion criteria, followed by data extraction and a quality assessment of the included studies using the Cochrane Collaboration's tool. Correlations between exercise and cardiopulmonary function indices [pulse wave velocity, respiratory exchange ratio, and peak oxygen uptake (VO_2_ peak)] were then evaluated using mean differences and 95% confidence intervals. All meta-analyses were conducted using R 3.12 software. Finally, five eligible studies involving 179 CKD patients were included. After intervention, a heterogeneity test showed that the VO_2_ peak values of the treatment group were greater than those of the control group, whereas no significant differences were found for the other indices. However, a sensitivity analysis showed inconsistent results both before and after intervention. Thus, we concluded that exercise might play an important role in improving the VO_2_ peak values in CKD patients. Additional studies are needed to verify this conclusion.

## 1. Introduction

Chronic kidney disease (CKD, also known as chronic renal disease) is defined as a progressive loss of kidney structure and function [1]. CKD is a public health problem, affecting 5%–10% of the world's population [2, 3]; CKD caused 956,000 deaths in 2013, which is more than twice the number recorded in 1990 [4]. Based on disease severity, CKD is classified into five stages, with stages 1–4 considered as early stages and 5 as the advanced stage [5, 6]. CKD is typically associated with an increase in urine protein or serum creatinine levels and can result from diabetes mellitus, glomerulonephritis, or hypertension [7]. In addition, the primary cause of death in CKD patients is cardiovascular disease, regardless of whether the patient has reached stage 5 [8–10].

Water-based exercise improves oxidative stress status, renal function, and cardiorespiratory function in patients with moderate renal failure and can be used by patients with chronic renal failure, together with dietary modifications, blood pressure control, education, and encouragement, to delay renal atherosclerosis and cardiovascular complications and to prevent physical worsening [11]. Exercise training improves endurance, muscle strength, and maximal exercise capacity in predialysis CKD patients, showing positive effects on muscle catabolism, health-related quality of life, and functional capacity [12]. Castaneda et al. [13] investigated the influences of resistance training on inflammatory mediators (interleukin-6 and serum C-reactive protein) and nutritional status in 26 adult CKD patients. These authors found that resistance training improved nutritional status and decreased inflammation in CKD patients on a low-protein diet. Toyama et al. [14] explored the therapeutic effects of exercise in 19 patients with both cardiovascular disease (CVD) and CKD. Measurements of estimated glomerular filtration rate (eGFR), high-density lipoprotein-cholesterol (HDL-C) levels, anaerobic metabolic threshold (AT) VO_2_ values, and triglyceride levels revealed that exercise therapy improved renal function in patients with both CVD and CKD by improving lipid metabolism. Balakrishnan et al. [15] determined that resistance exercise increased skeletal muscle mitochondrial DNA copy number in 23 CKD patients. Heiwe and Jacobson [16] conducted a meta-analysis to examine the effects of exercise on health outcomes (aerobic capacity, cardiovascular function, health-related quality of life, muscular function, and walking capacity) in CKD patients, concluding that regular exercise training improved health outcomes in such patients.

Although the above studies focused on the effects of exercise on CKD patients, the sample sizes were small and/or cardiopulmonary function indices were incomplete. Thus, we performed the current meta-analysis to comprehensively assess the effects of exercise on cardiopulmonary function indices in CKD patients, including pulse wave velocity (PWV), respiratory exchange ratio (RER), and peak oxygen uptake (VO_2_ peak).

## 2. Materials and Methods

### 2.1. Search Strategy

A literature review was performed by searching literatures in PubMed and Embase before June 2016. In particular, the PubMed search strategy was as follows: #1 “exercise,” #2 “aerobic exercise,” #3 Search “resistance training,” #4 “#1 OR #2 OR #3,” #5 “chronic kidney disease,” #6 “CKD,” #7 “chronic nephropathy,” #8 “chronic renal disease,” #9 “#5 OR #6 OR #7 OR #8,” #10 “random^*∗*^,” #11 “#4 and #9 and #10.” The Embase search strategy was as follows: (“exercise”/exp OR “aerobic exercise”/exp OR “resistance training”/exp) AND (“chronic kidney disease”/exp OR “chronic nephropathy”/exp OR “chronic renal disease”/exp) AND random^*∗*^.

### 2.2. Inclusion and Exclusion Criteria

The inclusion criteria were as follows: (1) a study that focused on the association between exercise and cardiopulmonary function indices (such as artery stiffness, PWV, and VO_2_ peak) in CKD patients; (2) a randomized controlled trial in which the treatment and control groups comprised CKD patients with and without exercise management, respectively; and (3) a study in which data on artery stiffness, PWV, and/or VO_2_ peak values were available or could be calculated. Reviews, reports, letters, and comments were excluded.

### 2.3. Data Extraction and Quality Assessment

Two reviewers independently selected the eligible studies and extracted the following data: the name of the first author, the year of publication, the study location, the duration of follow-up, patient CKD stage, patient demographics [including sex ratio, age range, body mass index (BMI), and eGFR], and cardiopulmonary function indices (including PWV, VO_2_ peak, and RER) of the CKD patients in the treatment and control groups. Afterward, a quality assessment of the eligible studies was performed using the Cochrane Collaboration's tool to evaluate the risk of bias [17]. During data extraction and quality assessment, any disagreement between the two investigators was settled by discussion with a third reviewer until a consensus was reached.

### 2.4. Statistical Analysis

R 3.12 software (R Foundation for Statistical Computing, Beijing, China, meta package) was used for this meta-analysis. The mean differences (MDs) and their 95% confidence intervals (95% CIs) were used to evaluate the correlations between exercise and cardiopulmonary function indices of the CKD patients. A heterogeneity test for the studies was based on the *Q*-test [18] and *I*^2^-statistic [19]. When heterogeneity was significant (*P* < 0.05 or *I*^2^ > 50%), the random effects model (REM) was used to pool the sizes of the effect. However, when heterogeneity was insignificant (*P* ≥ 0.05 and *I*^2^ ≤ 50%), the fixed effects model was used [20]. According to Cochrane's suggestion, publication bias [21] can be verified using Egger's method if more than 10 studies are included. Finally, a sensitivity analysis was performed by ignoring one study at a time to evaluate its effects on the pooled MDs [22].

## 3. Results

### 3.1. Eligible Studies

**Figure 1:**
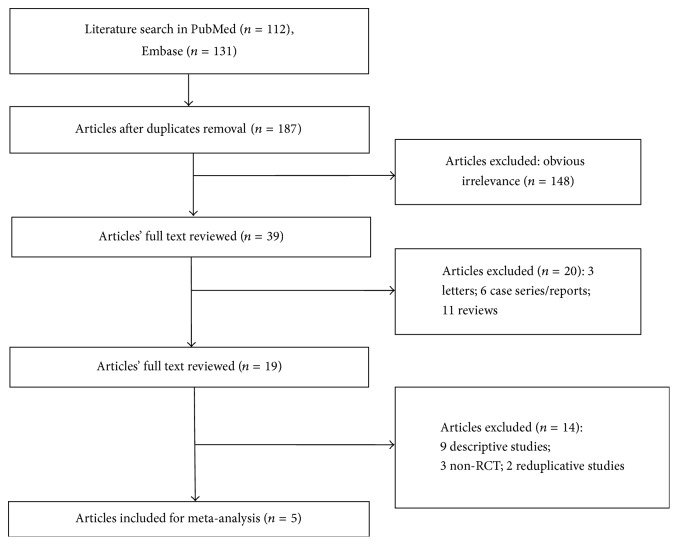
Flow chart of the literature review.

The flow chart of the literature review is found in Figure 1. According to the predefined search strategy, a total of 243 studies were initially obtained from the Embase and PubMed databases. After removing 56 duplicates, 187 studies remained. After browsing the titles and abstracts, 148 studies were eliminated as irrelevant. Thirty-four studies (including 11 reviews, 3 letters, 6 case series/reports, and 9 descriptive researches) were further excluded after reading the full text. Five studies were finally included in the present meta-analysis [23–27].

**Figure 2:**
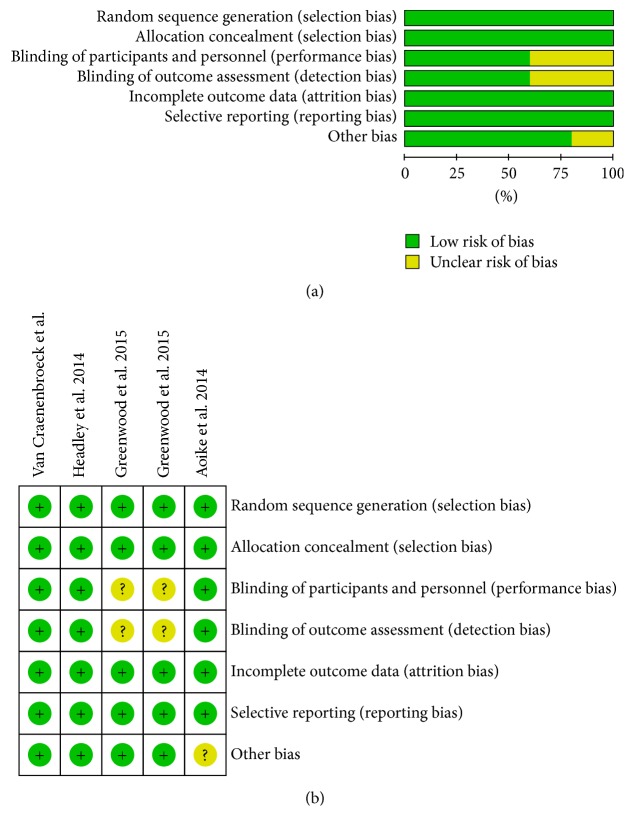
Quality assessment of the included studies. (a) Bias risk of the eligible studies. (b) Sensitivity and specificity of the eligible studies. “?” represents an unclear risk of bias; “+” indicates a low risk of bias.

The detailed characteristics of the included studies are listed in Table 1. The five eligible studies involved 179 CKD patients: 93 in the treatment group and 86 in the control group. The studies were published from 2014 to 2015, and the patients mainly showed stage 3/4 CKD and were located in Brazil, the United States, and Belgium. Sex distribution among the two groups was consistent, with the number of men higher than that of women. The two groups mainly comprised middle-aged and elderly individuals, with no significant differences in age. There were no significant differences in BMI or eGFR between the two groups, and the average BMI was larger than 24. In addition, the follow-up duration ranged from 12 weeks to 1 year (Figure 2).

### 3.2. Correlation between Exercise and Cardiopulmonary Function Indices in CKD Patients

**Figure 3:**
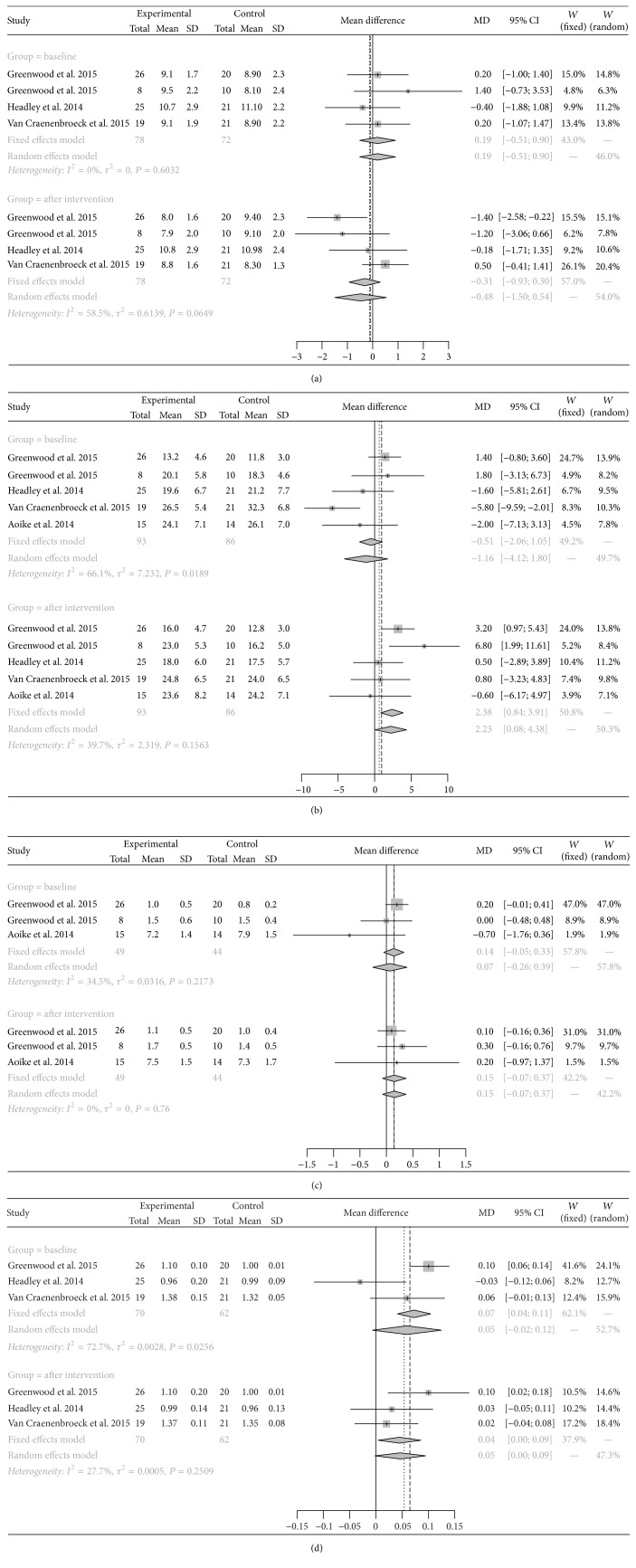
Forest plots of correlations between exercise and pulse wave velocity (a), peak oxygen uptake (mL/kg/min, (b)), peak oxygen uptake (L/min, (c)), and respiratory exchange ratio (d) in patients with chronic kidney disease.

The PWV, VO_2_ peak values, and RERs of the CKD patients in the two groups were analyzed both before and after intervention. Significant heterogeneity was found among studies examining VO_2_ peak values (*I*^2^ = 66.1%; *P* = 0.019) and those examining RERs (*I*^2^ = 72.7%; *P* = 0.026) before intervention, whereas heterogeneity was only present in studies examining PWV (*I*^2^ = 58.5%; *P* = 0.065) after intervention. However, because only five studies were included in the present meta-analysis, REM was employed for all analyses using the DerSimonian-Laird approach, which is the default method in the R package. There were no differences in the pooled results for each of the four indices between the two groups at baseline, whereas, after intervention, significantly higher VO_2_ peak values (MD: 2.23; 95% CI: 0.08–4.38) and RERs (MD: 0.05; 95% CI: 0.00–0.09) were found in the experimental group compared with those in the control group (Figure 3).

### 3.3. Publication Bias and Sensitivity Analysis

Because fewer than 10 studies were included, publication bias was not verified. A sensitivity analysis was performed for PWV, VO_2_ peak values, and RERs. The pooled MDs were not affected by ignoring any of the studies before, but not after, the intervention.

## 4. Discussion

The current meta-analysis was designed to comprehensively explore the association between exercise and cardiopulmonary function indices in CKD patients. Five eligible studies involving 179 CKD patients (93 patients in the treatment group and 86 patients in the control group) were included in the present meta-analysis. After intervention, pooled results showed that the VO_2_ peak values and RERs in the treatment group were higher than those in the control group, indicating that exercise significantly improved cardiopulmonary function in CKD patients.

Among the studies included in this meta-analysis, Aoike et al. [23] investigated the association between home-based aerobic training and cardiopulmonary and functional capacities in overweight non-dialysis-dependent (NDD) CKD patients. These authors found that exercise significantly increased cardiopulmonary (including VO_2_ peak) and functional capacities and decreased diastolic and systolic blood pressures. Greenwood et al. [24] evaluated the effects of exercise on eGFR, cardiorespiratory fitness, and vascular health in CKD patients, observing significant between-group MDs in creatinine-based eGFR levels, VO_2_ peak values, PWV, and waist circumference. Greenwood et al. [25] also examined the effects of resistance or aerobic training on indices of cardiovascular risk and vascular health in kidney transplant recipients, demonstrating that resistance and aerobic training were both beneficial in this population based on the significant improvement in VO_2_ peak values and PWV. Headley et al. [26] found that short-term, moderate-intensity exercise training did not change arterial stiffness but increased VO_2_ peak values in stage 3 CKD patients. Van Craenenbroeck et al. [27] reported that aerobic exercise training improved the quality of life and VO_2_ peak values in stage 3/4 CKD patients, without affecting arterial stiffness or endothelial function. Although cardiopulmonary function indices have not been comprehensively studied, all these studies indicated that exercise significantly improved VO_2_ peak values in CKD patients.

VO_2_ peak values are believed to be promising predictors of survival in patients with advanced renal disease [28]. Sakkas et al. [29] demonstrated that 6 months of cycle ergometry training improved the vascularization of the gastrocnemius muscle and increased maximum VO_2_ values in patients with chronic renal failure. In addition, patients with CKD and end-stage renal disease showed enhanced exercise tolerance and improved uremia symptoms after exercise training, with VO_2_ peak values increasing by 50%–70% [30]. In renal transplant and hemodialysis patients, muscle strength and VO_2_ peak values were found to increase significantly during exercise training [31]. In studies with a 1-year follow-up, NDD-CKD patients who performed home-based aerobic and resistance exercise showed increased VO_2_ peak values [32, 33]. In kidney transplant recipients, long-term training may be required to increase arterial venous oxygen levels and improve muscle metabolism and VO_2_ peak values [34]. In CKD patients, regular exercise can reduce blood pressure and improve VO_2_ peak values, muscular strength, quality of life, and physical performance, indicating that exercise might be essential for the successful treatment of such patients [35, 36].

This study used a meta-analysis to analyze the impact of exercise on cardiopulmonary function indices in CKD patients. We found that exercise significantly improved VO_2_ peak values in CKD patients. Nevertheless, there are several limitations of this study. First, the results of our meta-analysis might be affected by unknown sources of heterogeneity. Second, the meta-analysis and regression analysis of the subgroups were not conducted for age and race because the demographic characteristics in the included studies were incomplete. Third, a qualitative and quantitative verification of publication bias was not performed due to the small number of included studies. Finally, a sensitivity analysis showed that a fraction of our results before and after intervention was inconsistent, which might be due to the small number of eligible studies. Despite these limitations, a quality assessment showed that our results are reliable.

## 5. Conclusions

Exercise significantly improves VO_2_ peak values in patients with CKD. Additional high-quality studies are needed to verify this finding.

## Figures and Tables

**Table 1:** Detailed characteristics of the included studies.

First author	Publication year	Location	Follow-up	CKD Stage	Group	*n*	Age	Sex (male)	BMI/weight (kg)	eGFR (mL/min/1.73 m^2^)
Greenwood	2015	UK	12 weeks	NA	Aerobic training	13	53.9 ± 10.7	10	26.6 ± 4.7	49.0 ± 18.1
					Resistance training	13	54.6 ± 10.6	7	28.2 ± 3.6	48.3 ± 12.4
					Usual-care group	20	49.5 ± 10.6	10	27.3 ± 3.6	47.1 ± 16.2
Headley	2014	USA	16 weeks	Stage 3	Treatment	25	58.0 ± 8.0	16	101.7 ± 24.9	47.0 ± 12.0
					Control	21	57.1 ± 9.0	14	104.8 ± 29.8	48.3 ± 12.7
Van Craenenbroeck	2015	Belgium	3 months	Stages 3 and 4	Exercise training	19	51.5 ± 11.8	11	28.3 ± 6.2	37.5 ± 13.23
					Usual care	21	54.7 ± 14.1	11	28.3 ± 5.8	39.6 ± 12.9
Aoike	2014	Brazil	12 weeks	Stages 3 and 4	Home-based group	15	55.9 ± 7.7	10	31.7 ± 4.5	28.4 ± 11.2
					Control group	14	54.3 ± 8.7	9	30.7 ± 4.1	25.3 ± 13.4
Greenwood	2015	UK	12 months	Stages 3 and 4	Aerobic training	8	53.8 ± 13.5	6	27.40 ± 3.52	36.6 ± 10.1
					Usual-care group	10	53.3 ± 12.9	9	28.44 ± 4.24	46.5 ± 20.6

UK: United Kingdom; CKD: chronic kidney disease; BMI: body mass index; eGFR: estimated glomerular filtration rate.
